# Ovarian cancer risk, ALDH2 polymorphism and alcohol drinking: Asian data from the Ovarian Cancer Association Consortium

**DOI:** 10.1111/cas.13470

**Published:** 2018-01-21

**Authors:** Tomotaka Ugai, Linda E. Kelemen, Mika Mizuno, Jue‐Sheng Ong, Penelope M. Webb, Georgia Chenevix‐Trench, on behalf of the Australian Ovarian Cancer Study Group, Kristine G. Wicklund, Jennifer Anne Doherty, Mary Anne Rossing, Pamela J. Thompson, Lynne R. Wilkens, Michael E. Carney, Marc T. Goodman, Joellen M. Schildkraut, Andrew Berchuck, Daniel W. Cramer, Kathryn L. Terry, Hui Cai, Xiao‐Ou Shu, Yu‐Tang Gao, Yong‐Bing Xiang, David Van Den Berg, Malcom C Pike, Anna H. Wu, Celeste Leigh Pearce, Keitaro Matsuo, on behalf of the Ovarian Cancer Association Consortium

**Affiliations:** ^1^ Division of Molecular and Clinical Epidemiology Aichi Cancer Center Research Institute Nagoya Japan; ^2^ Division of Hematology Saitama Medical Center Jichi Medical University Saitama Japan; ^3^ Department of Public Health Sciences College of Medicine and Hollings Cancer Center Medical University of South Carolina Charleston USA; ^4^ Department of Gynecologic Oncology Aichi Cancer Center Hospital Nagoya Japan; ^5^ Genetics and Computational Biology Department QIMR Berghofer Medical Research Institute Brisbane Australia; ^6^ Population Health Department QIMR Berghofer Medical Research Institute Brisbane Australia; ^7^ Program in Epidemiology Division of Public Health Sciences Fred Hutchinson Cancer Research Center Seattle USA; ^8^ Huntsman Cancer Institute Population Health Sciences University of Utah Salt Lake City USA; ^9^ Department of Epidemiology University of Washington Seattle USA; ^10^ Cancer Prevention and Control Samuel Oschin Comprehensive Cancer Institute Cedars‐Sinai Medical Center Los Angeles USA; ^11^ Cancer Epidemiology Program University of Hawaii Cancer Center Honolulu USA; ^12^ Department of Obstetrics and Gynecology John A. Burns School of Medicine University of Hawaii Honolulu USA; ^13^ Community and Population Health Research Institute Department of Biomedical Sciences Cedars‐Sinai Medical Center Los Angeles USA; ^14^ Department of Public Health Sciences The University of Virginia Charlottesville USA; ^15^ Department of Obstetrics and Gynecology Duke University Medical Center Durham USA; ^16^ Obstetrics and Gynecology Epidemiology Center Brigham and Women's Hospital Boston USA; ^17^ Harvard T. H. Chan School of Public Health Boston USA; ^18^ Vanderbilt Epidemiology Center Vanderbilt University School of Medicine Nashville USA; ^19^ Department of Epidemiology Shanghai Cancer Institute Renji Hospital Shanghai Jiaotong University School of Medicine Shanghai China; ^20^ SKLORG & Department of Epidemiology Shanghai Cancer Institute Shanghai China; ^21^ Department of Preventive Medicine Keck School of Medicine University of Southern California Norris Comprehensive Cancer Center Los Angeles USA; ^22^ Department of Epidemiology and Biostatistics Memorial Sloan‐Kettering Cancer Center NY USA; ^23^ Department of Epidemiology University of Michigan School of Public Health Ann Arbor USA; ^24^ Department of Epidemiology Nagoya University Graduate School of Medicine Nagoya Japan

**Keywords:** *ALDH2*, Asian, drinking habit, ovarian cancer, pooled analysis

## Abstract

The aldehyde dehydrogenase 2 (*ALDH2*) polymorphism rs671 (Glu504Lys) causes ALDH2 inactivation and adverse acetaldehyde exposure among Asians, but little is known of the association between alcohol consumption and rs671 and ovarian cancer (OvCa) in Asians. We conducted a pooled analysis of Asian ancestry participants in the Ovarian Cancer Association Consortium. We included seven case‐control studies and one cohort study comprising 460 invasive OvCa cases, 37 borderline mucinous OvCa and 1274 controls of Asian descent with information on recent alcohol consumption. Pooled odds ratios (OR) with 95% confidence intervals (CI) for OvCa risk associated with alcohol consumption, rs671 and their interaction were estimated using logistic regression models adjusted for potential confounders. No significant association was observed for daily alcohol intake with invasive OvCa (OR comparing any consumption to none = 0.83; 95% CI = 0.58‐1.18) or with individual histotypes. A significant decreased risk was seen for carriers of one or both Lys alleles of rs671 for invasive mucinous OvCa (OR = 0.44; 95% CI = 0.20‐0.97) and for invasive and borderline mucinous tumors combined (OR = 0.48; 95% CI = 0.26‐0.89). No significant interaction was observed between alcohol consumption and rs671 genotypes. In conclusion, self‐reported alcohol consumption at the quantities estimated was not associated with OvCa risk among Asians. Because the rs671 Lys allele causes ALDH2 inactivation leading to increased acetaldehyde exposure, the observed inverse genetic association with mucinous ovarian cancer is inferred to mean that alcohol intake may be a risk factor for this histotype. This association will require replication in a larger sample.

## 1 INTRODUCTION

Ovarian cancer is one of the most common gynecological cancers. Approximately 239 000 females developed a new ovarian cancer in 2012 and 152 000 women died globally of the disease.^1^ Despite its high incidence and mortality, the etiology is not fully understood; however, established epidemiological risk factors for ovarian cancer include age, parity, oral contraceptive use, tubal ligation, and inherited germline mutations in *BRCA1* and *BRCA2*.^2^, ^3^

Alcohol consumption is one of the possible modifiable risk factors for ovarian cancer. Several studies have investigated the association between alcohol drinking and ovarian cancer risk and reported inconsistent results.^4^, ^5^, ^6^, ^7^, ^8^, ^9^, ^10^, ^11^, ^12^, ^13^, ^14^ To resolve this inconsistency, pooled analyses have been conducted.^5^, ^15^, ^16^, ^17^ These studies failed to show a clear association between alcohol drinking and ovarian cancer risk overall; however, some showed a different trend in associations with alcohol by histological subtypes,^16^, ^17^ suggesting different biological etiologies according to histology.^18^

Generally, a differential distribution pattern of the histological subtypes of epithelial ovarian cancer has been observed across ethnicities and countries.^19^ Among Asian women, the prevalence of serous adenocarcinoma is relatively low, whereas that of clear cell adenocarcinoma is higher, compared with ovarian cancers among women of European descent. Furthermore, Asian women are likely to have different genetic and sociocultural backgrounds, which includes less alcohol consumption,^20^ lower prevalence of hormone therapy use^21^ and a different distribution of the aldehyde dehydrogenase 2 (*ALDH2*) polymorphism Glu504Lys (rs671).^22^ The rs671 polymorphism in *ALDH2* is more prevalent in East‐Asian populations (minor allele frequency [MAF] in HapMap‐JPT = 0.24, and 0.15 in HapMap‐HCB)^22^ and absent among Europeans (MAF HapMap‐CEU = 0). The Lys allele of rs671 is strongly associated with inactivation of ALDH2,^23^, ^24^ which results in prolonged exposure to the intermediate metabolite acetaldehyde, a potential carcinogen in various organs.^25^, ^26^, ^27^, ^28^, ^29^, ^30^ To our knowledge, there are no studies exploring the association between rs671 in *ALDH2* and ovarian cancer risk, particularly among Asian women.

To investigate whether there is an association between alcohol drinking, the rs671 polymorphism in *ALDH2* and ovarian cancer risk, we conducted a pooled analysis of data from women of Asian ancestry participating in the Ovarian Cancer Association Consortium (OCAC).

## 2 MATERIALS AND METHODS

### 2.1 Study population

We conducted this pooled analysis using seven case‐control studies and one cohort study with information on alcohol consumption from the OCAC. We included 460 invasive ovarian cancer cases, 37 borderline mucinous tumors and 1274 controls. Other borderline tumors (n = 23) except mucinous were excluded from the analysis because, unlike other ovarian histotypes, mutational evidence suggests mucinous tumors progress along a multistep continuum from benign to borderline to invasive tumors.^31^

Information from the eight studies is summarized in Table 1. All study participants were of Asian ancestry in Japan [JPN^32^, ^33^], China [SWH^34^], Australia [AUS^35^], and the USA [DOV,^36^ HAW,^37^ NCO,^38^, ^39^ NEC,^40^, ^41^ and USC^42^]. One study was a hospital‐based study, six were population‐based studies, and one was a defined cohort study. Informed consent was obtained from participating subjects in each of the individual studies, and local human research investigations committees approved each study. This investigation was approved by a human research investigations committee at Aichi Cancer Center.

**Table 1:** List of participating studies and number of subjects

Study acronym [reference #]	Study name	Country	Study design	Controls, N	Invasive cases, N	Borderline cases, N	Lys allele frequency among invasive cases (%)	Lys allele frequency among controls (%)	Median age (range), invasive cases	Median age (range), controls	Proportion of ever drinkers among cases (%)	Proportion of ever drinkers among controls (%)
				1274	460	59	18.8	22.5	54 (23‐85)	52.2 (19‐88)	18.7	10.5
AUS^35^	Australia Ovarian Cancer Study & Australia Cancer Study (AOCS/ACS)	Australia	Population‐based	16	26	0	9.6	21.9	48.5 (31‐63)	49 (23‐79)	42.3	31.3
DOV^36^	Diseases of the Ovary and their Evaluation (DOVE)	USA	Population‐based	41	40	10	12.5	8.5	48.5 (35‐74)	50 (36‐69)	24.0	36.6
HAW^37^	Hawaii Ovarian Cancer Study	USA	Population‐based	204	103	18	19.9	19.1	59 (28‐85)	58 (22‐88)	14.1	37.8
JPN^32^, ^33^	Hospital‐based Epidemiologic Research Program at Aichi (HERPACC)	Japan	Hospital‐based	81	67	11	29.1	29.0	5 4(23‐75)	53 (19‐74)	36.7	40.7
NCO^38^, ^39^	North Carolina Ovarian Cancer Study (NCOCS)	USA	Population‐based	5	6	5	0.0	30.0	41.5 (24‐61)	56 (43‐73)	72.7	40.0
NEC^40^, ^41^	New England‐based Case‐Control Study of Ovarian Cancer (NECC)	USA	Population‐based	6	11	6	13.6	8.3	39 (27‐61)	39.5 (34‐61)	47.1	50.0
SWH^34^	Shanghai Women's Health Study (SWHS)	China	Defined cohort	864	135	0	20.4	23.8	57.2 (43‐81)	51.6 (40‐71)	3.0	2.55
USC^42^	Los Angeles County Case‐Control Studies of Ovarian Cancer (LAC‐CCOC)	USA	Population‐based	57	72	9	13.9	15.8	49 (23‐84)	47 (24‐78)	9.9	5.26

### 2.2 Genotyping methods

Genotyping was carried out as part of the Collaborative Oncological Gene‐environment Study (COGS),^43^ a collaboration between the OCAC and three other consortia. Full details of selection of single nucleotide polymorphisms (SNP), array design, genotyping and post‐genotyping quality control have been described elsewhere.^44^ SNP on the iCOGS chip were categorized into three categories: (i) selected on the basis of pooled genome‐wide association study data; (ii) selected for the fine‐mapping of published risk loci; and (iii) selected on the basis of previous analyses or specific hypotheses. SNP rs671 on ALDH2 was a candidate SNP selected on the basis of specific hypotheses described above.

For the OCAC samples, genotyping of 211 155 SNP in 47 630 samples from 43 individual studies was conducted using a custom Illumina Infinium array (iCOGS; Illumina, San Diego, CA, USA) across two centers, of which 44 308 passed quality control. Genotypes were called using Illumina's proprietary GenCall algorithm. Standard quality control measures were applied across all SNP and all samples. Samples were excluded for any of the following reasons: genotypically not female XX (XY, XXY or XO); overall call rate <95%; low or high heterozygosity (*P* < 10^−6^); individuals not concordant with previous genotyping within the OCAC; individuals where genotypes for the duplicate sample appeared to be from a different individual; cryptic duplicates within studies where the phenotypic data indicated that the individuals were different, or between studies where genotype data indicated samples were duplicates; and samples from first‐degree relatives. We used the program LAMP^45^ to assign intercontinental ancestry on the basis of genotype frequencies in the European, Asian and African populations in OCAC samples. Individuals with >20% minority ancestry for the Asian ancestral group were considered mixed ancestry and excluded based on LAMP analysis. We then used a set of 37 000 unlinked markers to carry out principal components analysis within the Asian ancestral group to identify residual population substructure.^46^ For the analyses of Asian subjects, we included five principal components as covariates.

### 2.3 Alcohol assessment and covariate data collection

Harmonization of daily alcohol intake across OCAC studies was previously described.^16^ Briefly, daily alcohol consumption was estimated using validated food frequency questionnaires (FFQ) in AUS,^47^ DOV,^48^ HAW,^49^ NEC,^50^ SWH,^51^ and USC or from questions regarding alcohol intake embedded in a risk factor questionnaire (NCO, JPN). The exposure period was the year preceding recruitment (AUS, HAW, JPN, NEC, SWH, USC) or at the time period approximately 4 (DOV) or 5 (NCO) years before the reference date. Daily alcohol consumption in grams was determined by summing the product of frequency of consumption of specified alcoholic beverages (beer, wine, and other alcoholic beverages, including liquor, Japanese Sake, Chuuhai and Shochu) by the alcohol content of each beverage using national estimates of alcohol content for that country. Total alcohol intake was calculated as the sum of each alcohol intake and used for the analysis. The AUS, DOV, HAW, and NEC studies provided the information for white and red wine separately.

Key clinical, demographic and questionnaire data on study subjects (see below) were merged into a common dataset by the coordinating center and checked for consistency.

### 2.4 Data analyses

Differences in categorized demographic variables between the cases and controls were tested using the chi‐squared test except where there were a large number of missing observations.

To assess the strength of the associations of *ALDH2* polymorphism and daily alcohol consumption with the risk of invasive ovarian cancer, odds ratios (OR) with 95% confidence intervals (CI) were estimated using unconditional logistic regression models. The alcohol consumption analyses used as the reference group women who did not consume any type of alcoholic beverage. Based on the median value of grams per day of alcohol consumed (total alcohol and alcohol from beer, wine [white, red] and other alcohol) among controls (7.57 g/d), alcohol consumption was classified into two (none, any alcohol intake) and three categories (none, up to and including the median intake, more than the median intake). Models for the main effect of alcohol were adjusted for age, 5 Asian principal components, smoking status (never, ever smokers), and study. Missing values for covariates were treated as dummy variables in the models. Other possible confounders were excluded from the multivariate model as a result of a large number of missing observations. Risk models associated with total alcohol intake did not include other alcoholic beverage types. Risk models associated with beer, wine or liquor intake included all three beverage types and were thus adjusted for each other. Risk models associated with white or red wine intake included beer and liquor intake.

OR for the main effect of *ALDH2* genotypes on ovarian cancer risk were adjusted for age, 5 Asian principal components, and study under both codominant and dominant genetic models using the Glu/Glu genotype as reference. We conducted stratified analyses by histological subtypes and applied a multinomial logistic regression model to evaluate heterogeneity for an association of the *ALDH2* Lys allele across histological subtypes. Models were compared using the likelihood‐ratio test.

To assess the joint effect of genotype and alcohol intake, we created four categories combining genotype with alcohol intake: non‐Lys allele carriers and no alcohol intake as a reference group; non‐Lys allele carriers and any alcohol intake; Lys allele carriers and no alcohol intake; and Lys allele carriers and any alcohol intake.

Even though all study participants were of Asian ancestry, heterogeneity among studies might affect the results. Therefore, we repeated all analyses using random effects meta‐analyses to calculate summary study‐specific estimates.

*P*‐value <.05 was considered statistically significant. All analyses were carried out using STATA version 13.1 (Stata Corp., College Station, TX, USA).

## 3 RESULTS

**Figure 1:**
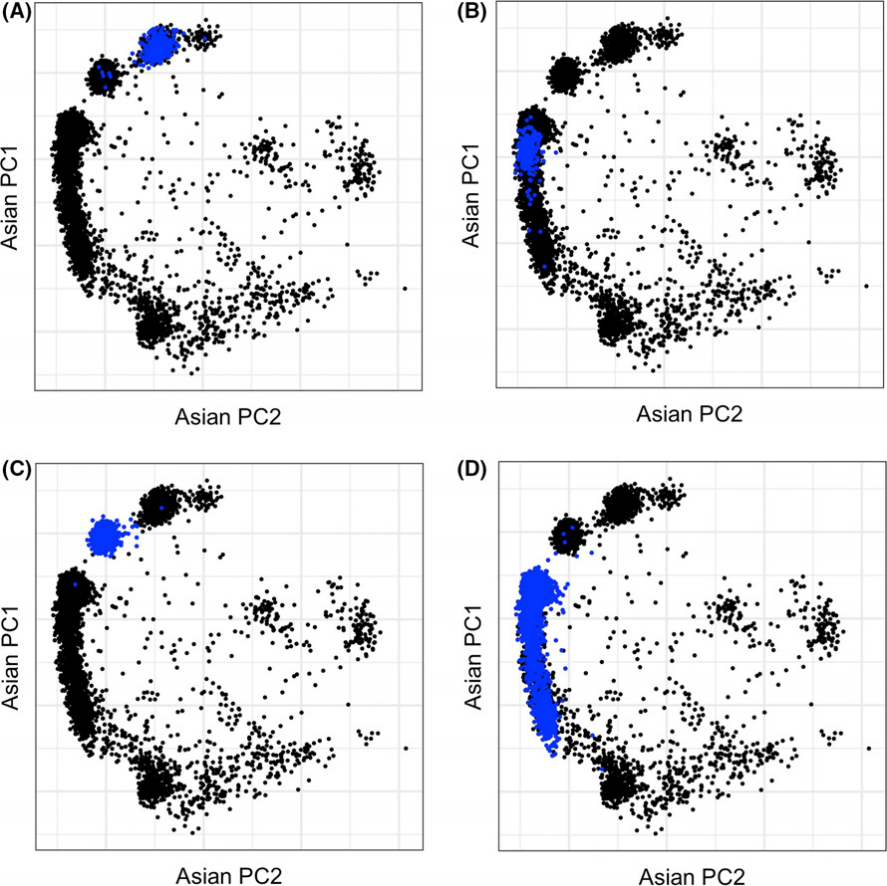
Genetic ancestry of Asians in Ovarian Cancer Association Consortium (OCAC) studies conducted in Asian countries. Plot of the first 2 principal components from each Asian ancestry participant from a single study (blue circles) superimposed over the first 2 principal components from all Asian ancestry participants that were genotyped in OCAC (black circles). The black circles take the form of countries denoting participants with ancestrally similar allele frequencies. A, JPN (Japan). B, SWH (Shanghai, China). C, KRA (Korea). D, CHA (China)

**Figure 2:**
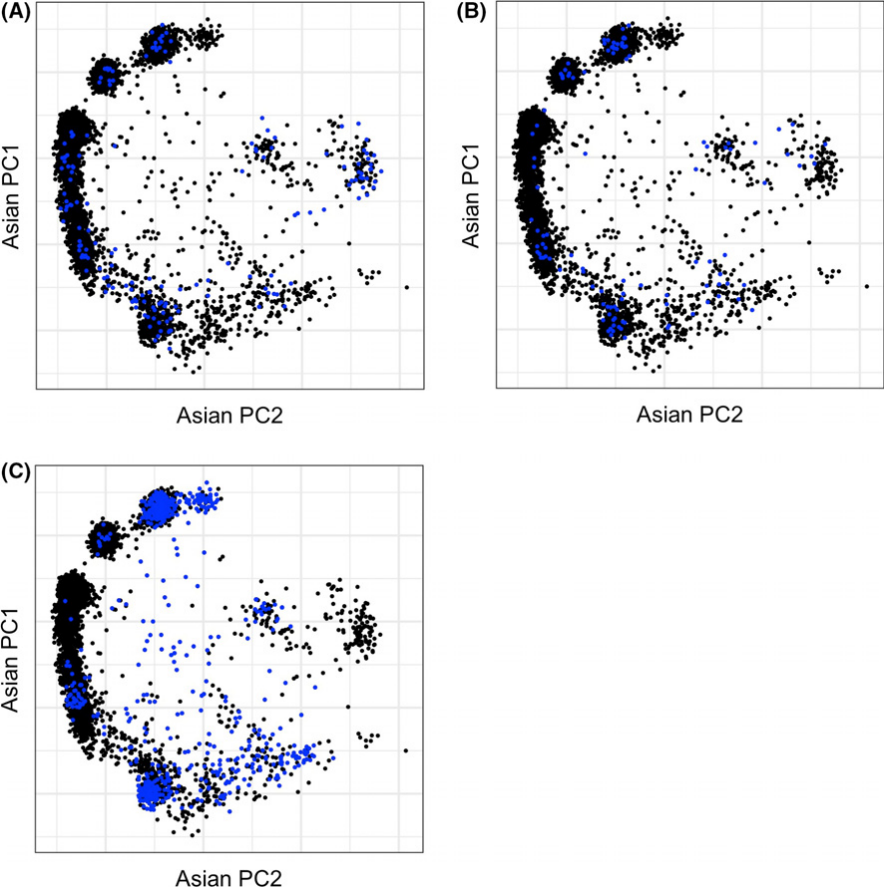
Genetic ancestry of Asians in Ovarian Cancer Association Consortium (OCAC) studies conducted in the USA. Plot of the first 2 principal components from each Asian ancestry participant from a single study (blue circles) superimposed over the first 2 principal components from all Asian ancestry participants that were genotyped in OCAC (black circles). Ancestral membership of Asian participants in the US studies can be mapped to country of origin. A, USC (California). B, DOV (Washington). C, HAW (Hawaii)

Table 1 shows the distribution of cases and controls, Lys allele frequency, median age and the proportion of ever drinkers for each study. Median age of cases and controls and Lys allele frequency varied across the eight studies with NEC showing the lowest allele frequency of 8.3% and NCO having the highest at 30% among controls. This reflects the diverse composition of participants categorized as “Asian” in these studies (eg, Chinese, Japanese, Korean or Pilipino). However, the two studies conducted in Asian countries (JPN and SWH) had relatively similar Lys allele frequencies (29% and 23.8%, respectively). To illustrate, the figures show the results from superimposing the data from the first two orthogonal principal components from over 30 000 unlinked markers from each Asian ancestry study participant from a single study (blue circles) onto the data from all Asian ancestry study participants in OCAC (black circles), and where the black clusters segregated according to country of genetic origin. In Figure 1, Asian participants from the two Asian countries, JPN (Japan) and SWH (Shanghai, China), are shown in panels A and B and participants from two other Asian studies, KRA (Korea) and CHA (China), are shown in panels C and D. Figure 2 shows that Asian participants from the USC (California) and DOV (Washington) studies in the USA (panels A and B) had allele frequencies mapping to regions in Japan, China and the Philippines, whereas Asian participants from the HAW (Hawaii) study had allele frequencies mapping more strongly and, not surprisingly, to regions in Japan and the Philippines and to a lesser extent to China. Subsequent statistical models controlled for this variability with the inclusion of five principal components as covariates. The proportion of ever drinkers was lower in SWH and USC, compared with other studies.

Demographic characteristics and selected lifestyle habits of study subjects are shown in Table 2. Distribution of histological subtypes among invasive ovarian cancer cases was 188 serous (40.9%), 42 mucinous (9.1%), 75 endometrioid (16.3%), and 69 clear cell (15.0%) adenocarcinomas. Overall, prevalence of the Lys allele carrier was 33.9% of cases and 39.5% of controls. Median total alcohol intake among controls who consumed alcohol recently was 7.57 g/d. Cases were more likely to drink alcohol (*P* < .001). The proportion of ever smokers was higher among cases. Overall, the median age of cases and controls was 54.0 and 52.2 years, respectively. A higher proportion of cases compared to controls was observed in the youngest and oldest age groups. Distribution of other variables (age at menarche, use of oral contraception, tubal ligation, low parity, body mass index [BMI], history of any prior cancer and family history of breast or ovarian cancer in first‐degree relatives) is shown in Table 2 but should be interpreted cautiously because of the large amount of missing data for both cases and controls.

**Table 2:** Characteristics of study subjects with invasive ovarian cancer

	Invasive cases (N = 460) (%)	Controls (N = 1274) (%)	*P*‐value^a^
Histology			
Serous	188 (40.9)		
Mucinous	42 (9.1)		
Endometrioid	75 (16.3)		
Clear cell	69 (15.0)		
Other epithelial	78 (17.0)		
Non‐epithelial	8 (1.7)		
*ALDH2* rs671 genotype			
Glu/Glu	304 (66.1)	771 (60.5)	.070
Glu/Lys	139 (30.2)	433 (34.0)	
Lys/Lys	17 (3.7)	70 (5.5)	
Total alcohol (grams per day)			
None	372 (80.9)	1135 (89.1)	<.001
0.1‐7.6	54 (11.7)	67 (5.3)	
7.6‐192.6	28 (6.1)	67 (5.3)	
Unknown	6 (1.3)	5 (0.4)	
Smoking status			
Never (%)	369 (80.2)	1133 (88.9)	.051
Ever (%)	54 (11.7)	118 (9.3)	
Unknown (%)	37 (8.0)	23 (1.8)	
Age (y)			
Median (range)	54.0 (23‐85)	52.2 (19‐88)	
<40 (%)	43 (9.4)	44 (3.5)	<.001
40‐49 (%)	106 (23.0)	480 (37.7)	
50‐59 (%)	154 (33.5)	379 (29.8)	
60‐69 (%)	99 (21.5)	282 (22.1)	
≥70 (%)	58 (12.6)	89 (7.0)	
Education			
Less than college graduate (%)	123 (26.7)	157 (12.3)	NE
More than college graduate (%)	98 (21.3)	149 (11.7)	
Unknown (%)	239 (52.0)	968 (76.0)	
Body mass index			
Median (range)	22.8 (16.7‐39.8)	22.4 (16.4‐34.0)	
<25 kg/m^2^ (%)	134 (29.1)	130 (10.2)	NE
≥25 kg/m^2^ (%)	42 (9.1)	32 (2.5)	
Unknown (%)	284 (61.7)	1112 (87.3)	
Age at menarche (y)			
Median (range)	13 (9‐21)	13 (9‐22)	
≤10 (%)	16 (3.5)	31 (2.4)	NE
11‐12 (%)	118 (25.7)	170 (13.3)	
13‐15 (%)	247 (53.7)	181 (14.2)	
≥16 (%)	78 (17.0)	26 (2.0)	
Unknown (%)	1 (0.2)	866 (68.0)	
Oral contraception			
Never (%)	216 (47.0)	222 (17.4)	NE
Ever (%)	107 (23.3)	186 (14.6)	
Unknown (%)	137 (29.8)	866 (68.0)	
Tubal ligation			
Yes (%)	42 (9.1)	68 (5.3)	NE
No (%)	215 (46.7)	261 (20.5)	
Unknown (%)	203 (44.1)	945 (74.2)	
Parity			
0 (%)	94 (20.4)	67 (5.3)	NE
1‐2 (%)	150 (32.6)	212 (16.6)	
≥3 (%)	79 (17.2)	129 (10.1)	
Unknown (%)	137 (29.8)	866 (68.0)	
History of any prior cancers			
No (%)	437 (95.0)	389 (30.5)	NE
Yes (%)	19 (4.1)	21 (1.7)	
Unknown (%)	4 (0.9)	864 (67.8)	
History of breast or ovarian cancer in first‐degree relatives			
No (%)	104 (22.6)	104 (8.2)	NE
Yes (%)	40 (8.7)	53 (4.2)	
Unknown (%)	316 (68.7)	1117 (87.7)	

a Chi‐squared test was performed except where there were a large number of missing observations.

NE, not estimated.

Table 3 presents the association between daily alcohol intake and invasive ovarian cancer risk in the Asian population adjusting for age, smoking status, study and principal components. OR associated with total alcohol intake of 0‐7.6 g/d and 7.6‐192.6 g/d among all ovarian cancers were 0.92 (95% CI = 0.59‐1.45) and 0.69 (95% CI = 0.42‐1.14), respectively (trend *P* = .188). No significant associations were observed for type of alcoholic beverage consumed. Analyses that adjusted for several covariates listed in Table 2 showed similar trends (data not shown). In addition, we carried out analyses excluding younger subjects, non‐drinkers, or Lys/Lys genotype, but none of the results was substantially altered (data not shown).

**Table 3:** Association between alcoholic beverage and invasive ovarian cancer risk among Asian population

	Cases (N = 460) / Controls (N = 1274)	OR (95% CI)	*P*‐value
Total alcohol (g/d)^a^			
None	372/1135	1 (ref.)	
0‐7.6	54/67	0.92 (0.59‐1.45)	.731
7.6‐192.6	28/67	0.69 (0.42‐1.14)	.148
Unknown	6/5	NE	
Beer (g/d)^b^			
None	372/1135	1 (ref.)	
0.2‐5.3	21/32	0.84 (0.41‐1.72)	.637
5.3‐136.9	18/32	1.01 (0.50‐2.04)	.724
Unknown	49/75	NE	
Wine (g/d)^b^			
None	372/1135	1 (ref.)	
0.1‐3.2	28/30	0.79 (0.36‐1.75)	.560
3.2‐192.6	20/43	0.70 (0.32‐1.51)	.360
Unknown	40/66	NE	
White wine (g/d)^c^^,^ d			
None	372/1135	1 (ref.)	
0.2‐3.2	15/20	0.60 (0.20‐1.80)	.358
3.2‐192.6	11/32	0.61 (0.24‐1.54)	.299
Unknown	62/87	NE	
Red wine (g/d)^c^^,^ d			
None	372/1135	1 (ref.)	
0.2‐3.1	17/28	0.45 (0.16‐1.24)	.124
3.1‐92.9	9/22	0.60 (0.22‐1.64)	.321
Unknown	62/89	NE	
Other alcoholic beverage (g/d)^b^^.^ e			
None	372/1135	1 (ref.)	
0.2‐7.5	21/26	0.97 (0.46‐2.06)	.939
7.5‐95.8	8/25	0.73 (0.28‐1.94)	.531
Unknown	59/88	NE	

a Odds ratios (OR) are adjusted for age, smoking, principal component 1‐5 and study site for total alcohol.

b OR for beer, wine, and other alcoholic beverages are mutually adjusted in addition to age, smoking, principal component 1‐5 and study site.

c OR for red wine and white wine are adjusted for beer and other alcoholic beverages in addition to age, smoking, principal component 1‐5 and study site.

d Including AUS, DOV, HAW, and NEC.

e Including liquor, Japanese sake, Chuuhai and Shochu.

NE, not estimated.

AUS, Australia Ovarian Cancer Study & Australia Cancer Study (AOCS/ACS); DOV, Diseases of the Ovary and their Evaluation (DOVE); HAW, Hawaii Ovarian Cancer Study; NEC, New England‐based Case‐Control Study of Ovarian Cancer (NECC).

Table 4 presents the effect of *ALDH2* rs671 genotypes and total alcohol intake on invasive ovarian cancer risk overall in the Asian population. No significant association between rs671 genotypes in *ALDH2* and invasive ovarian cancer risk overall was observed (OR for dominant model = 0.92; 95% CI = 0.71‐1.18; *P* = .490). No significant interaction between any alcohol consumption and rs671 in *ALDH2* was observed (interaction *P* = .634).

**Table 4:** Odds ratios of invasive ovarian cancer by ALDH2 genotype and alcohol intake according to histological subtype

	ALDH2 genotype^a^				Total alcohol^a^^,^ c		Interaction
	Glu/Glu	Glu/Lys	Lys/Lys	Glu/Lys+Lys/Lys	None	Any	*P*‐value*
Overall invasive tumor							
Cases/Controls	304/771	139/433	17/70	156/503	372/1135	82/134	.634
OR (95% CI)	1 (ref.)	0.96 (0.74‐1.24)	0.72 (0.41‐1.27)	0.92 (0.71‐1.18)	1 (ref.)	0.83 (0.58‐1.18)	
Serous Invasive							
Cases/Controls	125/771	57/433	6/70	63/503	154/1135	31/134	.962
OR (95% CI)	1 (ref.)	0.97 (0.67‐1.41)	0.55 (0.22‐1.37)	0.91 (0.63‐1.30)	1 (ref.)	0.68 (0.41‐1.12)	
Mucinous Invasive							
Cases/Controls	33/771	8/433	1/70	9/503	32/1135	9/134	NE
OR (95% CI)	1 (ref.)	0.45 (0.20‐1.04)	0.35 (0.04‐2.76)	**0.44 (0.20‐0.97)**	1 (ref.)	1.36 (0.53‐3.44)	
Mucinous (invasive + borderline)							
Cases/Controls	62/771	15/433	2/70	17/503	59/1135	18/134	.382
OR (95% CI)	1 (ref.)	**0.49 (0.26‐0.93)**	0.42 (0.09‐1.89)	**0.48 (0.26‐0.89)**	1 (ref.)	0.80 (0.40‐1.58)	
Endometrioid invasive							
Cases/Controls	50/771	23/433	2/70	25/503	60/1135	14/134	.741
OR (95% CI)	1 (ref.)	1.11 (0.64‐1.92)	0.58 (0.13‐2.53)	1.04 (0.61‐1.76)	1 (ref.)	0.61 (0.29‐1.27)	
Clear cell invasive							
Cases/Controls	39/771	24/433	6/70	30/503	53/1135	15/134	.659
OR (95% CI)	1 (ref.)	1.25 (0.71‐2.21)	1.78 (0.67‐4.74)	1.33 (0.77‐2.27)	1 (ref.)	0.76 (0.38‐1.52)	

*Interaction between ALDH2 genotype (Glu/Glu vs Glu/Lys+Lys/Lys) and any alcohol.

Odds ratios (OR) are adjusted for age, principal component 1‐5, and study site.

a OR are adjusted for age, smoking, principal component 1‐5, and study site.

Drinking amount of 6 cases and 5 controls are unknown.

Bold denotes statistical significance.

NE, not estimated.

Table 4 also presents associations between genotype and alcoholic intake stratified by histological subtype. The Lys allele was significantly inversely associated with both invasive mucinous (OR for dominant model = 0.44; 95% CI = 0.20‐0.97; *P* = .041) and invasive plus borderline mucinous tumors (OR in dominant model = 0.48; 95% CI = 0.26‐0.89; *P* = .018). We also included alcohol intake as a covariate in this model, but none of the results was substantially altered (invasive mucinous tumor: OR for dominant model = 0.46; 95% CI = 0.21‐1.04; *P* = .062, invasive plus borderline mucinous tumors: OR for dominant model = 0.46; 95% CI = 0.25‐0.85; *P* = .014). The test for heterogeneity for the association of the *ALDH2* Lys allele between the histological subtypes was not significant (*P*‐value for heterogeneity test = .20). There was no significant association between alcoholic intake and ovarian cancer for any of the histological subtypes. The OR associated with any alcohol intake were less than 1 with the exception of invasive mucinous cancer. There was no significant interaction with alcohol consumption with any of the associations (Table S1).

We also carried out meta‐analyses to calculate summary study‐specific estimates (Tables [Link], [Link], [Link], [Link]). Overall, the results did not change substantially, but the mucinous tumor cases were too few to calculate a study‐specific OR, and thus some studies were not included in the meta‐analyses.

## 4 DISCUSSION

In the present study, we did not observe significant associations between total alcohol intake and invasive ovarian cancer risk in Asian populations. We found that the Lys allele of rs671 was associated with a decreased risk of both invasive mucinous ovarian cancer and invasive plus borderline mucinous ovarian cancers, but not the other histotypes, although the test for heterogeneity was not significant. No significant interactions were observed between rs671 genotypes in *ALDH2* and alcohol intake with risk of invasive ovarian cancer.

Results from epidemiological studies investigating the association between alcohol drinking and ovarian cancer risk among Caucasians are inconsistent, reporting either a null association,^7^, ^8^, ^9^, ^10^, ^11^, ^12^ a positive association,^13^, ^14^ or negative associations.^4^, ^5^, ^6^ Alcohol has been hypothesized to induce carcinogenesis by increasing the circulating level of estrogens,^52^ oxidative stress, acetaldehyde, or depletion of folate.^53^ In contrast, alcohol is reported to have a protective potential against ovarian carcinogenesis by decreasing follicle stimulating hormone, luteinizing hormone and gonadotropin levels. Polyphenols contained in red wine were proposed to explain the inverse association observed between red wine and risk of ovarian cancer.^5^, ^6^, ^10^, ^54^ We did not observe any statistically significant associations between alcohol intake and ovarian cancer in the Asian participants in our study. The evidence to support a role of alcohol in ovarian cancer epidemiology in Asian populations is scarce and may warrant additional evaluation in larger studies.

The present analysis also examined ovarian cancer risk using the functional *ALDH2* rs671 polymorphism. The Lys allele acts as dominant negative, because the variant form can suppress the activity of the Glu allele by the formation of heterotetramers.^24^ Overall, 37.6% of our study subjects were heterozygous or homozygous for the null variant of *ALDH2* rs671. Inactive ALDH2 results in prolonged exposure to the metabolite, acetaldehyde, following alcohol intake. Peak blood acetaldehyde concentrations post alcohol challenge are 18 times and 5 times higher among homozygous null variant and heterozygous individuals compared with homozygous wild‐type individuals.^55^ This renders the consumption of alcohol unpleasant through inducing facial flushing, palpitations, drowsiness and other symptoms. Consequently, the *ALDH2* rs671 genotype has been used as a surrogate for alcohol consumption in studies using the Mendelian Randomization approach^56^, ^57^ because its interpretation is not influenced by confounding or bias that affects the interpretation of self‐reported alcohol intake. Therefore, it would be expected that carriers of the Lys allele (null variant), which associates with low alcohol intake, would be at lower risk of ovarian cancer, which is what was observed in the current study for invasive mucinous ovarian cancer and for combined invasive and borderline mucinous cancer (OR = 0.48, *P *= .018). This implies that alcohol consumption may be associated with increased risk of mucinous ovarian cancer.

The strengths of this investigation include the analysis of individual‐level data from a relatively large sample compared to previous studies, which allowed us to quantify risk associations of the *ALDH2* polymorphism, detailed drinking status and ovarian cancer risk. Other strengths are the uniform genotyping procedures and quality‐control measures adopted. We were also able to control for population stratification by first using LAMP analysis to identify Asian ancestral membership separate from other genetically similar groups, and then including 5 principal components as model covariates to control for residual genetic heterogeneity within the Asian membership.

The present study does have some weaknesses. The models for alcohol intake did not adjust for all potential confounders, because a substantial number of subjects from a single study (SWH) had missing values for several covariates. Further, the self‐reported alcohol quantities were either too low or measured with error and may have obscured an association with ovarian cancer if it existed whereas the genetic models are not influenced by these limitations. Despite the common prevalence of the *ALDH2* polymorphism among Asians, the small sample sizes for the histological type analysis precludes a conclusive interpretation of the results for Mendelian Randomization, which must await further study with a larger sample size. Finally, we did not adjust for multiple comparisons and a cautious interpretation of the histologically specific results is required.

In conclusion, we observed an inverse association between the Lys allele of rs671 in *ALDH2* and mucinous ovarian cancer risk in an Asian population. Because the rs671 Lys allele causes ALDH2 inactivation leading to increased acetaldehyde exposure, the observed inverse genetic association with mucinous ovarian cancer is inferred to mean that alcohol intake may be a risk factor for this histotype. Future investigation using even larger epidemiological studies of Asians is warranted.

## CONFLICTS OF INTEREST

Authors declare no conflicts of interest for this article.
