# Supplementary material for: Ovarian cancer risk, ALDH2 polymorphism and alcohol drinking: Asian data from the Ovarian Cancer Association Consortium
Source: Cancer Sci. 2018 Jan 21;109(2):435–45. doi: 10.1111/cas.13470 (PMC5797830; doi:10.1111/cas.13470)
Supplement: Supplementary file 1 [file CAS-109-435-s001.docx]

**Table S1. Interaction between ALDH2 genotype and alcohol intake according to the histological subtype**

|  |  | **ALDH2 genotype*Total alcohol** | | | |  | **Interaction** |
| --- | --- | --- | --- | --- | --- | --- | --- |
|  |  | Glu/Glu+None | Glu/Glu+Any | (Glu/Lys+Lys/Lys)+None | (Glu/Lys+Lys/Lys)+Any |  | ***P**** |
|  |  |  |  |  |  |  |  |
| **Overall invasive tumor** |  |  |  |  |  |  |  |
| Cases /Controls |  | 230/659 | 70/108 | 142/476 | 12/26 |  |  |
| OR (95%CI)† |  | 1 (ref.) | 0.83 (0.56-1.24) | 0.90 (0.69-1.18) | 0.62 (0.29-1.32) |  | 0.634 |
|  |  |  |  |  |  |  |  |
| **Serous Invasive** |  |  |  |  |  |  |  |
| Cases /Controls |  | 98/659 | 12/108 | 56/476 | 5/26 |  |  |
| OR (95%CI)† |  | 1 (ref.) | 0.65 (0.37-1.12) | 0.83 (0.56-1.22) | 0.55 (0.19-1.59) |  | 0.962 |
|  |  |  |  |  |  |  |  |
| **Mucinous Invasive** |  |  |  |  |  |  |  |
| Cases /Controls |  | 23/659 | 9/108 | 9/476 | 0/26 |  |  |
| OR (95%CI)† |  | 1 (ref.) | 1.50 (0.56-4.01) | 0.54 (0.24-1.25) | NE |  | NE |
|  |  |  |  |  |  |  |  |
| **Mucinous (invasive + borderline)** | |  |  |  |  |  |  |
| Cases /Controls |  | 43/659 | 17/108 | 16/476 | 1/26 |  |  |
| OR (95%CI)† |  | 1 (ref.) | 0.73 (0.35-1.54) | **0.49 (0.26-0.95)** | 0.13 (0.01-1.17) |  | 0.382 |
|  |  |  |  |  |  |  |  |
| **Endometrioid invasive** |  |  |  |  |  |  |  |
| Cases /Controls |  | 37/659 | 12/108 | 23/476 | 2/26 |  |  |
| OR (95%CI)† |  | 1 (ref.) | 0.64 (0.29-1.45) | 1.04 (0.59-1.85) | 0.50 (0.10-2.50) |  | 0.741 |
|  |  |  |  |  |  |  |  |
| **Clear cell invasive** |  |  |  |  |  |  |  |
| Cases /Controls |  | 26/659 | 12/108 | 27/476 | 3/26 |  |  |
| OR (95%CI)† |  | 1 (ref.) | 0.90 (0.40-2.03) | 1.36 (0.74-2.50) | 0.87 (0.22-3.51) |  | 0.659 |

Bold denotes statistical significance.

† ORs are adjusted for age, smoking, principle component 1-5, and study site.

*** Interaction between ALDH2 genotype (Glu/Glu vs Glu/Lys+Lys/Lys) and any alcohol**

***Abbreviations: OR*** odds ratio, ***NE*** not estimated.
