# Supplementary material for: Ovarian cancer risk, ALDH2 polymorphism and alcohol drinking: Asian data from the Ovarian Cancer Association Consortium
Source: Cancer Sci. 2018 Jan 21;109(2):435–45. doi: 10.1111/cas.13470 (PMC5797830; doi:10.1111/cas.13470)
Supplement: Supplementary file 2 [file CAS-109-435-s002.docx]

**Table S2. Association between alcoholic beverage and invasive ovarian cancer risk (Pooled analysis and meta-analysis)**

| **Total alcohol (grams per day)** |  | **Cases (N=460) / controls (N=1274)** | **OR (95% CI)†** |  | ***P* value** |
| --- | --- | --- | --- | --- | --- |
| **Original analysis (Pooled analysis)** | |  |  |  |  |
| None |  | 372/1135 | 1 (ref.) |  |  |
| 0-7.6 |  | 54/67 | 0.92 (0.59-1.45) |  | 0.731 |
| 7.6-192.6 |  | 28/67 | 0.69 (0.42-1.14) |  | 0.148 |
| Unknown |  | 6/5 | - |  |  |
|  |  |  |  |  |  |
| **Meta-analysis** |  |  |  |  |  |
| None |  | 372/1135 | 1 (ref.) |  |  |
| 0-7.6 |  | 49/65 | 0.93 (0.56-1.53) |  | 0.781 |
| 7.6-192.6 |  | 27/67 | 0.59 (0.21-1.69) |  | 0.325 |
| Unknown |  | 6/5 | - |  |  |
|  |  |  |  |  |  |
| **AUS** |  |  |  |  |  |
| None |  | 12/4 | 1 (ref.) |  |  |
| 0-7.6 |  | 10/8 | 0.45 (0.06-3.50) |  | 0.444 |
| 7.6-192.6 |  | 1/4 | 0.01 (0.0004-0.35) |  | 0.011 |
| Unknown |  | 3/0 | - |  |  |
|  |  |  |  |  |  |
| **DOV** |  |  |  |  |  |
| None |  | 28/23 | 1 (ref.) |  |  |
| 0-7.6 |  | 8/11 | 0.55 (0.16-1.85) |  | 0.333 |
| 7.6-192.6 |  | 2/4 | 0.33 (0.04-2.59) |  | 0.294 |
| Unknown |  | 2/3 | - |  |  |
|  |  |  |  |  |  |
| **HAW** |  | HAW |  |  |  |
| None |  | 89/155 | 1 (ref.) |  |  |
| 0-7.6 |  | 7/13 | 0.89 (0.33-2.42) |  | 0.822 |
| 7.6-192.6 |  | 7/36 | 0.33 (0.14-0.79) |  | 0.013 |
| Unknown |  | 0/0 | - |  |  |
|  |  |  |  |  |  |
| **JPN** |  |  |  |  |  |
| None |  | 41/49 | 1 (ref.) |  |  |
| 0-7.6 |  | 17/21 | 1.21 (0.54-2.73) |  | 0.640 |
| 7.6-192.6 |  | 9/11 | 1.01 (0.34-3.01) |  | 0.986 |
| Unknown |  | 0/0 | - |  |  |
|  |  |  |  |  |  |
| **NCO** |  |  |  |  |  |
| None |  | 1/3 | 1 (ref.) |  |  |
| 0-7.6 |  | 5/2 | NE |  |  |
| 7.6-192.6 |  | 0/0 | NE |  |  |
| Unknown |  | 0/0 | - |  |  |
|  |  |  |  |  |  |
| **NEC** |  |  |  |  |  |
| None |  | 6/3 | 1 (ref.) |  |  |
| 0-7.6 |  | 3/3 | NE |  | 0.954 |
| 7.6-192.6 |  | 1/0 | NE |  |  |
| Unknown |  | 1/1 | - |  |  |
|  |  |  |  |  |  |
| **SWH** |  |  |  |  |  |
| None |  | 131/846 | 1 (ref.) |  |  |
| 0-7.6 |  | 2/8 | 1.18 (0.22-6.42) |  | 0.846 |
| 7.6-192.6 |  | 2/10 | 1.47 (0.29-7.41) |  | 0.639 |
| Unknown |  | 0/0 | - |  |  |
|  |  |  |  |  |  |
| **USC** |  |  |  |  |  |
| None |  | 64/53 | 1 (ref.) |  |  |
| 0-7.6 |  | 2/1 | 1.57 (0.13-19.3) |  | 0.726 |
| 7.6-192.6 |  | 6/2 | 3.40 (0.51-22.4) |  | 0.204 |
| Unknown |  | 0/1 | - |  |  |
|  |  |  |  |  |  |

† ORs are adjusted for age, smoking, principle component 1-5 and study site for total alcohol.

***Abbreviations: OR*** odds ratio, ***NE*** not estimated.
