# Supplementary material for: Ovarian cancer risk, ALDH2 polymorphism and alcohol drinking: Asian data from the Ovarian Cancer Association Consortium
Source: Cancer Sci. 2018 Jan 21;109(2):435–45. doi: 10.1111/cas.13470 (PMC5797830; doi:10.1111/cas.13470)
Supplement: Supplementary file 3 [file CAS-109-435-s003.docx]

**Table S3. Odds ratios of invasive ovarian cancer by ALDH2 genotype and alcohol intake (Pooled analysis and meta-analysis)**

|  |  | **ALDH2 genotype†** | | | |  | **Total alcohol‡, §** | |
| --- | --- | --- | --- | --- | --- | --- | --- | --- |
|  |  | Glu/Glu | Glu/Lys | Lys/Lys | Glu/Lys+Lys/Lys |  | None | Any |
| **Overall invasive tumor (pooled analysis)** | | |  |  |  |  |  |  |
| Cases /Controls |  | 304/771 | 139/433 | 17/70 | 156/503 |  | 372/1135 | 82/134 |
| OR (95%CI) |  | 1 (ref.) | 0.96 (0.74-1.24) | 0.66 (0.36-1.19) | 0.92 (0.71-1.18) |  | 1 (ref.) | 0.83 (0.58-1.18) |
|  |  |  |  |  |  |  |  |  |
| **Overall invasive tumor (meta-analysis)** | | |  |  |  |  |  |  |
| Cases /Controls |  | 298/766 | 139/432 | 17/69 | 156/501 |  | 371/1132 | 77/132 |
| OR (95%CI) |  | 1 (ref.) | 0.97 (0.64-1.27) | 0.73 (0.38-1.41) | 0.94 (0.72-1.22) |  | 1 (ref.) | 0.78 (0.45-1.35) |
| **AUS** |  |  |  |  |  |  |  |  |
| Cases /Controls |  | 22/10 | 3/5 | 1/1 | 4/6 |  | 12/4 | 11/12 |
| OR (95%CI) |  | 1 (ref.) | 0.16 (0.02-1.71) | 0.71 (0.02-20.0) | 0.23 (0.03-1.89) |  | 1 (ref.) | 0.18 (0.03-1.08) |
|  |  |  |  |  |  |  |  |  |
| **DOV** |  |  |  |  |  |  |  |  |
| Cases /Controls |  | 31/35 | 8/5 | 1/1 | 9/6 |  | 28/23 | 10/15 |
| OR (95%CI) |  | 1 (ref.) | 1.51 (0.39-5.82) | 1.37 (0.07-25.2) | 1.49 (0.41-5.33) |  | 1 (ref.) | 0.50 (0.16-1.55) |
|  |  |  |  |  |  |  |  |  |
| **HAW** |  |  |  |  |  |  |  |  |
| Cases /Controls |  | 65/137 | 35/56 | 3/11 | 38/67 |  | 89/155 | 14/49 |
| OR (95%CI) |  | 1 (ref.) | 1.29 (0.75-2.21) | 0.50 (0.13-1.93) | 1.16 (0.69-1.96) |  | 1 (ref.) | 0.48 (0.24-0.94) |
|  |  |  |  |  |  |  |  |  |
| **JPN** |  |  |  |  |  |  |  |  |
| Cases /Controls |  | 34/40 | 27/35 | 6/6 | 33/41 |  | 41/49 | 26/32 |
| OR (95%CI) |  | 1 (ref.) | 0.81 (0.40-1.65) | 1.09 (0.31-3.83) | 0.85 (0.43-1.67) |  | 1 (ref.) | 1.14 (0.56-2.33) |
|  |  |  |  |  |  |  |  |  |
| **NCO** |  |  |  |  |  |  |  |  |
| Cases /Controls |  | 6/3 | 0/1 | 0/1 | 0/2 |  | 1/3 | 5/2 |
| OR (95%CI) |  | 1 (ref.) | NE | NE | NE |  | 1 (ref.) | NE |
|  |  |  |  |  |  |  |  |  |
| **NEC** |  |  |  |  |  |  |  |  |
| Cases /Controls |  | 1/5 | 3/1 | 0/0 | 3/1 |  | 6/2 | 4/3 |
| OR (95%CI) |  | 1 (ref.) | 0.83 (0.02-38.8) | NE | 0.83 (0.02-38.8) |  | 1 (ref.) | 1.67 (0.01-531.5) |
|  |  |  |  |  |  |  |  |  |
| **SWH** |  |  |  |  |  |  |  |  |
| Cases /Controls |  | 84/501 | 47/314 | 4/49 | 51/363 |  | 131/846 | 4/18 |
| OR (95%CI) |  | 1 (ref.) | 0.92 (0.61-1.39) | 0.47 (0.15-1.43) | 0.86 (0.58-1.28) |  | 1 (ref.) | 1.32 (0.41-4.28) |
|  |  |  |  |  |  |  |  |  |
| **USC** |  |  |  |  |  |  |  |  |
| Cases /Controls |  | 54/40 | 16/16 | 2/1 | 18/17 |  | 64/53 | 8/3 |
| OR (95%CI) |  | 1 (ref.) | 0.89 (0.37-2.14) | 2.81 (0.23-34.8) | 0.97 (0.41-2.29) |  | 1 (ref.) | 2.64 (0.57-12.3) |

Bold denotes statistical significance.

† ORs are adjusted for age, principle component 1-5, and study site.

‡ ORs are adjusted for age, smoking, principle component 1-5, and study site.

§ Drinking amount of six cases and five controls are unknown.

***Abbreviations: OR*** odds ratio, ***NE*** not estimated.
