# Supplementary material for: Ovarian cancer risk, ALDH2 polymorphism and alcohol drinking: Asian data from the Ovarian Cancer Association Consortium
Source: Cancer Sci. 2018 Jan 21;109(2):435–45. doi: 10.1111/cas.13470 (PMC5797830; doi:10.1111/cas.13470)
Supplement: Supplementary file 4 [file CAS-109-435-s004.docx]

**Table S4. Odds ratios of mucinous invasive cancer by ALDH2 genotype and alcohol intake (Pooled analysis and meta-analysis)**

|  |  | **ALDH2 genotype†** | | | |  | **Total alcohol‡, §** | |
| --- | --- | --- | --- | --- | --- | --- | --- | --- |
|  |  | Glu/Glu | Glu/Lys | Lys/Lys | Glu/Lys+Lys/Lys |  | None | Any |
| **Mucinous Invasive (pooled analysis)** | |  |  |  |  |  |  |  |
| Cases /Controls |  | 33/771 | 8/433 | 1/70 | 9/503 |  | 32/1135 | 9/134 |
| OR (95%CI) |  | 1 (ref.) | 0.45 (0.20-1.04) | 0.35 (0.04-2.76) | **0.44 (0.20-0.97)** |  | 1 (ref.) | 1.36 (0.53-3.44) |
|  |  |  |  |  |  |  |  |  |
| **Mucinous Invasive (meta-analysis)** | |  |  |  |  |  |  |  |
| Cases /Controls |  | 27/678 | 6/386 | 1/11 | 7/447 |  | 19/208 | 6/52 |
| OR (95%CI) |  | 1 (ref.) | 0.46 (0.17-1.23) | 0.78 (0.07-8.92) | 0.44 (0.17-1.13) |  | 1 (ref.) | 0.91 (0.24-3.47) |
| **AUS** |  |  |  |  |  |  |  |  |
| Cases /Controls |  | 1/10 | 0/5 | 0/1 | 0/6 |  | 0/4 | 1/12 |
| OR (95%CI) |  | 1 (ref.) | NE | NE | NE |  | 1 (ref.) | NE |
|  |  |  |  |  |  |  |  |  |
| **DOV** |  |  |  |  |  |  |  |  |
| Cases /Controls |  | 0/35 | 0/5 | 0/1 | 0/6 |  | 0/23 | 0/15 |
| OR (95%CI) |  | 1 (ref.) | NE | NE | NE |  | 1 (ref.) | NE |
|  |  |  |  |  |  |  |  |  |
| **HAW** |  |  |  |  |  |  |  |  |
| Cases /Controls |  | 10/137 | 2/56 | 1/11 | 3/67 |  | 10/155 | 3/49 |
| OR (95%CI) |  | 1 (ref.) | 0.37 (0.07-2.03) | 0.78 (0.07-8.92) | 0.44 (0.10-1.99) |  | 1 (ref.) | 0.66 (0.15-2.83) |
|  |  |  |  |  |  |  |  |  |
| **JPN** |  |  |  |  |  |  |  |  |
| Cases /Controls |  | 2/40 | 0/35 | 0/6 | 0/41 |  | 0/49 | 2/32 |
| OR (95%CI) |  | 1 (ref.) | NE | NE | NE |  | 1 (ref.) | NE |
|  |  |  |  |  |  |  |  |  |
| **NCO** |  |  |  |  |  |  |  |  |
| Cases /Controls |  | 2/3 | 0/1 | 0/1 | 0/2 |  | 0/3 | 2/2 |
| OR (95%CI) |  | 1 (ref.) | NE | NE | NE |  | 1 (ref.) | NE |
|  |  |  |  |  |  |  |  |  |
| **NEC** |  |  |  |  |  |  |  |  |
| Cases /Controls |  | 1/5 | 2/1 | 0/0 | 2/1 |  | 2/2 | 0/3 |
| OR (95%CI) |  | 1 (ref.) | NE | NE | NE |  | 1 (ref.) | NE |
|  |  |  |  |  |  |  |  |  |
| **SWH** |  |  |  |  |  |  |  |  |
| Cases /Controls |  | 8/501 | 3/314 | 0/49 | 3/363 |  | 11/846 | 0/18 |
| OR (95%CI) |  | 1 (ref.) | 0.61 (0.15-2.40) | NE | 0.49 (0.12-1.95) |  | 1 (ref.) | NE |
|  |  |  |  |  |  |  |  |  |
| **USC** |  |  |  |  |  |  |  |  |
| Cases /Controls |  | 9/40 | 1/16 | 0/1 | 1/17 |  | 9/53 | 1/3 |
| OR (95%CI) |  | 1 (ref.) | 0.33 (0.03-3.35) | NE | 0.33 (0.03-3.31) |  | 1 (ref.) | 3.67 (0.17-81.8) |

Bold denotes statistical significance.

† ORs are adjusted for age, principle component 1-5, and study site.

‡ ORs are adjusted for age, smoking, principle component 1-5, and study site.

§ Drinking amount of six cases and five controls are unknown.

***Abbreviations: OR*** odds ratio, ***NE*** not estimated.
