# Supplementary material for: Ovarian cancer risk, ALDH2 polymorphism and alcohol drinking: Asian data from the Ovarian Cancer Association Consortium
Source: Cancer Sci. 2018 Jan 21;109(2):435–45. doi: 10.1111/cas.13470 (PMC5797830; doi:10.1111/cas.13470)
Supplement: Supplementary file 5 [file CAS-109-435-s005.docx]

**Table S5. Odds ratios of mucinous (invasive + borderline) cancer by ALDH2 genotype and alcohol intake (Pooled analysis and meta-analysis)**

|  |  | **ALDH2 genotype†** | | | |  | **Total alcohol‡, §** | |
| --- | --- | --- | --- | --- | --- | --- | --- | --- |
|  |  | Glu/Glu | Glu/Lys | Lys/Lys | Glu/Lys+Lys/Lys |  | None | Any |
| **Mucinous (invasive + borderline) / Pooled analysis** | | |  |  |  |  |  |  |
| Cases /Controls |  | 62/771 | 15/433 | 2/70 | 17/503 |  | 59/1135 | 18/134 |
| OR (95%CI) |  | 1 (ref.) | 0.49 (0.26-0.93) | 0.42 (0.09-1.89) | **0.48 (0.26-0.89)** |  | 1 (ref.) | 0.80 (0.40-1.58) |
|  |  |  |  |  |  |  |  |  |
| **Mucinous (invasive + borderline) / Meta-analysis** | | |  |  |  |  |  |  |
| Cases /Controls |  | 52/753 | 12/426 | 2/12 | 14/494 |  | 43/280 | 11/99 |
| OR (95%CI) |  | 1 (ref.) | 0.49 (0.23-1.01) | 1.01 (0.14-7.53) | 0.47 (0.23-0.96) |  | 1 (ref.) | 0.79 (0.33-1.90) |
|  |  |  |  |  |  |  |  |  |
| **AUS** |  |  |  |  |  |  |  |  |
| Cases /Controls |  | 1/10 | 0/5 | 0/1 | 0/6 |  | 0/4 | 1/12 |
| OR (95%CI) |  | 1 (ref.) | NE | NE | NE |  | 1 (ref.) | NE |
|  |  |  |  |  |  |  |  |  |
| **DOV** |  |  |  |  |  |  |  |  |
| Cases /Controls |  | 5/35 | 1/5 | 1/1 | 2/6 |  | 5/23 | 1/15 |
| OR (95%CI) |  | 1 (ref.) | 1.28 (0.09-18.2) | 4.31 (0.15-127.0) | 1.91 (0.21-17.4) |  | 1 (ref.) | 0.04 (0.001-2.41) |
|  |  |  |  |  |  |  |  |  |
| **HAW** |  |  |  |  |  |  |  |  |
| Cases /Controls |  | 17/137 | 4/56 | 1/11 | 5/67 |  | 17/155 | 5/49 |
| OR (95%CI) |  | 1 (ref.) | 0.46 (0.13-1.64) | 0.49 (0.05-4.90) | 0.47 (0.14-1.53) |  | 1 (ref.) | 0.67 (0.21-2.10) |
|  |  |  |  |  |  |  |  |  |
| **JPN** |  |  |  |  |  |  |  |  |
| Cases /Controls |  | 6/40 | 3/35 | 0/6 | 3/41 |  | 5/49 | 4/32 |
| OR (95%CI) |  | 1 (ref.) | 0.50 (0.10-2.62) | NE | 0.42 (0.08-2.16) |  | 1 (ref.) | 1.15 (0.23-5.66) |
|  |  |  |  |  |  |  |  |  |
| **NCO** |  |  |  |  |  |  |  |  |
| Cases /Controls |  | 5/3 | 0/1 | 0/1 | 0/2 |  | 1/3 | 4/2 |
| OR (95%CI) |  | 1 (ref.) | NE | NE | NE |  | 1 (ref.) | NE |
|  |  |  |  |  |  |  |  |  |
| **NEC** |  |  |  |  |  |  |  |  |
| Cases /Controls |  | 4/5 | 3/1 | 0/0 | 3/1 |  | 4/2 | 2/3 |
| OR (95%CI) |  | 1 (ref.) | NE | NE | NE |  | 1 (ref.) | NE |
|  |  |  |  |  |  |  |  |  |
| **SWH** |  |  |  |  |  |  |  |  |
| Cases /Controls |  | 8/501 | 3/314 | 0/49 | 3/363 |  | 11/846 | 0/18 |
| OR (95%CI) |  | 1 (ref.) | 0.61 (0.15-2.40) | NE | 0.49 (0.12-1.95) |  | 1 (ref.) | NE |
|  |  |  |  |  |  |  |  |  |
| **USC** |  |  |  |  |  |  |  |  |
| Cases /Controls |  | 16/40 | 1/16 | 0/1 | 1/17 |  | 16/53 | 1/3 |
| OR (95%CI) |  | 1 (ref.) | 0.17 (0.02-1.44) | NE | 0.16 (0.02-1.39) |  | 1 (ref.) | 1.20 (0.10-14.8) |

Bold denotes statistical significance.

† ORs are adjusted for age, principle component 1-5, and study site.

‡ ORs are adjusted for age, smoking, principle component 1-5, and study site.

§ Drinking amount of six cases and five controls are unknown.

***Abbreviations: OR*** odds ratio, ***NE*** not estimate
